# Noninvasive Diagnosis of NAFLD and NASH

**DOI:** 10.3390/cells9041005

**Published:** 2020-04-17

**Authors:** Valeria Annarita Piazzolla, Alessandra Mangia

**Affiliations:** Liver Unit, Department of Medical Sciences, IRCCS Fondazione, “Casa Sollievo della Sofferenza”, 71013 San Giovanni Rotondo, Italy; valeria.piazzo@libero.it

**Keywords:** nonalcoholic steatohepatitis, nonalcoholic fatty liver disease, noninvasive tests, MRI, MRE, TE, OMICS

## Abstract

The aim of this review is to outline emerging biomarkers that can serve as early diagnostic tools to identify patients with nonalcoholic fatty liver disease (NAFLD) and nonalcoholic steatohepatitis (NASH) and, among them, the subgroup of best candidates for clinical trials on emerging compounds. Regarding possible predictors of NAFLD, a number of studies evaluated a combination of serum biomarkers either available in routine practice (or investigational) or proprietary and expensive. So far, magnetic resonance imaging-derived proton density fat fraction (MRI-PDFF) appears to be the most accurate for fatty liver diagnosis. In clinical practice, the main question is how to diagnose NASH early. There are new promising biomarkers that can help in diagnosing early stages of NASH, yet they include variables not routinely tested. In the setting of NASH, most studies confirm that, in spite of several well-known limitations, transient elastography or point shear wave elastography can help in enriching the pool of patients that should be screened for investigational treatments. Newer multiomics biomarkers including those focusing on microbiota can be useful but require methods to be standardized and implemented. To date, one biomarker alone is not able to non- or minimally invasively identify patients with NASH and mild to moderate fibrosis.

## 1. Introduction

Nonalcoholic fatty liver disease (NAFLD) represents a spectrum of chronic liver diseases on the rise worldwide, encompassing simple fatty liver, nonalcoholic steatohepatitis (NASH), and fibrosis. The presence of coexisting risk factors such as diabetes, metabolic syndrome, and obesity increases the risk of NAFLD. As a consequence of obesity pandemic and type 2 diabetes, an increased number of patients with NASH—the most severe form of NAFLD—is expected in the near future [1] Currently, no pharmacologic therapy is approved for treatment of NASH. The current standard treatment is lifestyle modification based on bodyweight and exercise. Novel therapeutic approaches are under clinical development.

Since both NAFLD and NASH are asymptomatic until advanced disease, many patients are only identified at advanced stages; thus, risk factor modification and current or experimental treatments are ineffective. Therefore, early predictors need to be investigated [2].

Liver biopsy is the current gold standard in diagnosis and prognosis; nevertheless, it is an expensive and invasive procedure with high sampling error and risk of complications including pain; bleeding; and, in very rare cases, death. Because of poor patient acceptance of this invasive standard technique, there is an urgent need for reliable, accurate, and non- or minimally invasive biomarkers.

A biomarker is a patient characteristic assessed as an indicator of a normal or a pathologic process or of a biological response to treatment. Unfortunately, to date, existing non- or minimally invasive biomarkers are inadequate.

While a number of non- or minimally invasive tests are able to rule out fibrosis or cirrhosis, no single test to identify steatosis, to early diagnose NASH, or to predict the disease progression is available. Moreover, specialized, combined tests are required to assess treatment response in clinical trials on emerging compounds.

Among minimally invasive tools, plasma biomarkers and composite scores defined as “wet biomarkers” and liver stiffness are commonly used. New biomarkers testing strategies such as imaging, procollagen-C3, and application of multiomics technologies are being developed. This review summarizes current biomarkers of NAFLD and NASH and explores candidate biomarkers under development.

## 2. Non- or Minimally Invasive Methods

Several methods (both proprietary and nonproprietary) have been suggested for minimally invasive quantification of hepatic fat and inflammation and for NASH diagnosis and assessment—including imaging and biomarker panels. However, no widely accepted reliable methods other than liver biopsy are yet available for non- or minimally invasive differentiation and risk estimation of simple steatosis and NASH in routine practice. Moreover, a major barrier for identification of subjects eligible for pharmacological intervention and enrolment in clinical trials is represented by the lack of non- or minimally invasive means of targeting those subjects undergoing liver biopsy that are likely to meet the histopathological criteria for NASH with fibrosis.

## 3. Non- or Minimally Invasive Differentiation of Simple Steatosis from NASH

### 3.1. Historical Biomarkers

Liver enzymes per se are not reliable and accurate predictors. Indeed, while incidentally abnormal liver enzymes are frequently reported in patients with NAFLD [3,4], liver enzymes may be normal in up to 80% of NAFLD patients [5]. Moreover, patients with advanced liver disease show decreased Alanine aminotransferase levels. [6,7]. Finally, in NAFLD patients with normal ALT, liver histology is not different from that of those with abnormal liver enzymes [8].

With improvements in technology, early studies on multiple circulating biomarkers including microRNA (miRNA) and cell-free nuclear material DNA or RNA show promising results. However, further work is needed before establishing their real diagnostic impact. For example, circulating miRNAs—small (18–24 nucleotides long) endogenous noncoding RNA molecules able to affect protein synthesis at the posttranscriptional level—are good markers of tissue status. Despite miRNAs, especially miR-122 and miR34a, possibly being considered promising diagnostic biomarkers for NAFLD—as they are linked to the lipid metabolism—they belong to an investigational area [9]. Combinations of several markers of steatosis at the aim of improving diagnostic performance have been evaluated.

The NAFLD Liver Fat Score (NLFS) evaluates the measurement of liver fat content and has shown satisfactory accuracy in diagnosing NAFLD. It is calculated based on metabolic syndrome, type 2 diabetes, fasting serum insulin, fasting serum aspartate aminotransferase/alanine aminotransferase ratio (AAR). In a cohort of 470 patients, a score greater than −0.640 predicted NAFLD with a sensitivity of 86% and a specificity of 71%. Using cutoff scores of −1.413 and ≥1.257, the sensitivity for the prediction of NAFLD is 95% (with 52% and 51% specificity, respectively) [10]. The inclusion of serum insulin level, which is not a routine test, in the formula might limit its wider clinical use.

The Hepatic Steatosis Index (HIS), which includes AST/ALT ratio, BMI, diabetes, and gender information, has been based on a very large cohort of Korean patients and validated against ultrasound that may be operator dependent. It showed a sensitivity of 66% and a specificity of 69% [11]. It seems to perform less well in diabetic patients.

The Fatty Liver Index (FLI) comprises BMI, waist circumference, and serum levels of triglycerides and gamma-glutamyltransferase (GGT) [12]. It has shown good performance in detecting fatty liver, although it has been validated against ultrasonography rather than liver histology.

In general population, these three tools offer a diagnostic efficacy of 70–80% with lower sensitivity and specificity in comparison to what was initially shown [13].

The VAI (Visceral Adiposity index) [14], a surrogate biomarker of visceral adiposity, and the TyG (triglyceride and Glucose index) [15] both have been shown independently associated with histologically defined steatosis in patients with Hepatitis C virus infection. In a head-to-head comparison, all the five indices reported above were shown accurate in diagnosing steatosis in comparison with the liver histology [16], but none of them can be used to quantitate liver fat and to consequently assess treatment response.

Commercial panels with more specialized parameters are also available to predict hepatic steatosis. Steato Test (Biopredictive, Paris, France) was constructed using a combination of the six components of Fibro Test-ActiTest (Biopredictive; comprising serum levels of total bilirubin, GGT, alfa-macroglobulin, haptoglobin, ALT, and apolipoprotein Al) plus BMI and serum levels of total cholesterol, triglycerides, and glucose adjusted for age and gender. [17]. This is a commercially available test and cannot be used in real-life practice. As the previously reported indexes, it cannot distinguish the grade of steatosis.

In summary, these fatty liver indices offer modest efficacy in detecting steatosis. They may help as surrogate parameters for liver fat content. Imaging techniques were shown more promising in steatosis assessment.

### 3.2. Emerging Noninvasive Imaging Methods for NAFLD Diagnosis

#### 3.2.1. Magnetic Resonance Imaging

Advanced magnetic resonance imaging (MRI) can measure the proton density fat fraction (PDFF), an objective and quantitative indicator of hepatic fat content across the entire liver, in an accurate, reproducible manner [18,19,20,21]. The ability to grade hepatic steatosis in adults with nonalcoholic steatohepatitis (NASH) and to minimize errors due to factors such as T1 bias, T2* decay, and the multifrequency signal interference effects of protons in fat that confound fat quantification with conventional MRI leads MRI-PDFF to be considered the leading tool for assessing longitudinal changes in hepatic fat [22]. Moreover, MRI-PDFF has been validated against liver histology and shown to be more sensitive in detecting changes in hepatic fat content and treatment response in clinical trials. In a study based on ezetimibe treatment, a reduction of 29% in liver fat on MRI-PDFF correlated with histological observations in NASH patients [23]. In a multi-center phase IIb study on 113 adult patients enrolled to obeticolic acid or placebo, using central histology as reference, steatosis was assessed at baseline and at treatment week 72 by MRI-PDFF at different sites. MRI-PDFF was shown to accurately classify grades and changes in hepatic steatosis [24].

#### 3.2.2. CAP

Transient elastography (TE) is a commercial ultrasound-based modality that measures liver stiffness as a surrogate for hepatic fibrosis using transient elastography. Controlled attenuation parameter (CAP) is a technique enabling the simultaneous measurement of stiffness and steatosis. Recent studies have shown that CAP significantly correlates with the percentage of steatosis and steatosis grade and that median CAP is higher among patients with significant steatosis. Significant relationship between the degree of steatosis and CAP (r = 0.81) was shown in a study published in 2012 [25,26]. Meyers et al. examined 153 overweight patients with chronic liver disease (47% with ascertained steatosis) and reported lower Area Under the Receiver Operating Characteristics (AUROC) values (0.79, 0.76 and 0.70, respectively) [27].

In a large study based on more than 5300 examinations, De Ledighen et al. found that CAP values were significantly associated with all parameters of metabolic syndrome [28]. In total, CAP has been studied in relatively few patients with biopsy proven NAFLD. In a study on 265 patients using both CAP and liver histology, De Ledighen reported AUROC 0.80 for steatosis grade ≥2 and 0.66 for steatosis grade 3 [28]. At a cutoff value of 310 deciBel per meter (dB/m), a positive predictive value for ≥S2 steatosis was 86% and the negative predictive value was 71%. Further histological correlation in a large-scale study is needed to establish diagnostic thresholds in NAFLD patients.

In summary, based on comparisons with liver histology, MRI-PDFF is emerging as the leading biomarker to assess liver fat. Baseline assessment and liver fat decline after investigational treatments can be accurately performed by MRI-PDFF. CAP evaluation may represent a first, easy-to-perform screening tool in the general population.

## 4. Methods for NASH Diagnosis

### 4.1. Panels for Diagnosis of NASH and Fibrosis Including Minimally Invasive Blood Markers

In a study from France on 125,052 NAFLD/NASH hospitalized patients, about 10% had compensated or decompensated cirrhosis at diagnosis. Moreover, NASH was associated with rapid progression and 27.5% of patients was associated with compensated disease progressed to decompensated cirrhosis over a period of 7 years [29]. This background emphasizes the need for minimally invasive serum tests for early detecting fibrosis associated with NASH. The performance of noninvasive biomarker of fibrosis needs to be compared with that of liver histology.

Although with a modest accuracy, plasma cytokeratin 18 (CK18) fragment levels, a marker of hepatocyte apoptosis, has been extensively evaluated in steatohepatitis [30]. At the investigational level, a positive association has been recently demonstrated between soluble macrophage activation marker CD163 (haemoglobin haptogobin scavenger receptor expressed exclusively on monocytes and macrophages) and histologically ascertained NASH fibrosis in two independent cohorts of NAFLD patients from Australia and Italy, suggesting a potential role for sCD 163 [31].

However, given that the accuracy of an individual marker—in addition to cytokeratin 18, inflammatory markers such as TNF and IL-8, and hormones such as adiponectin or FGF21—is modest, different diagnostic panels have been developed during the last 15 years, but their performances vary across studies.

Pelekar et al. [32] developed a panel that includes 6 variables: age, sex, AST, BMI, AST/ALT ratio, and serum Hyaluronic Acid. This model had an area under the receiver operating characteristic curve (AUROC) not higher than of 0.76.

Among proprietary testing, the NASHTest combines 13 biochemical and clinical variables (age; sex; height; weight; and serum levels of triglycerides (TGs), cholesterol, a-macroglobulin, apolipoprotein A1, haptoglobin, GGT, ALT, AST, and total bilirubin) to predict the presence or absence of NASH, achieving specificity, sensitivity, Positive predictive value (PPV), and negative predictive value (NPV) of 94%, 33%, 66%, and 81%, respectively, and (AUROC) of 0.79 [33,34,35]. This test requires several variables that are not routinely measured with consequent limitations of its use.

Fibrosis-4 index (Fib-4) was originally developed for HCV/HIV co-infected patients. Shah et al. validated its use in patients with NAFLD, comparing its performance with that of other markers [36]. The score is based on routinely available variables including age, ALT, AST, and platelets. AUROC for advanced fibrosis was 0.80 with positive and negative predictive values of 80% for FIB-4 score of 2.67 and of 90% for FIB-4 score of 1.30. In addition, this test can accurately exclude patients with F2 fibrosis.

The NASH Diagnostic panel was developed by Younoussi for predicting NASH. Patients with biopsy-proven NASH were evaluated by a model including diabetes, gender, BMI, triglycerides, and not routinely citokeratin markers such as M30 (apoptosis) and M65–M30 (necrosis) [37]. The same predictors were included in the NASH-related fibrosis prediction model (AUC: 0.80, 95% CI 0.68–0.88, 307 *p*-value < 0.00014). The NASH-related advanced fibrosis model includes type 2 diabetes, serum triglycerides, tissue inhibitor of metalloproteasea-1, and AST (AUC: 0.81, 95% CI, 0.70–0.89; *p*-value, 0.000062) [37].

More recently, a validated serum test to differentiate mild–moderate fibrosis (F0–F2) from advanced fibrosis (F3–F4) in NAFLD patients utilizing concentrations of alpha2 macrogobulin (A2M), HA, and TIMP-1 proteins was associated with an AUROC of 0.85 (95% CI, 0.820–0.892). It identified patients with 79.7% sensitivity (95% CI, 71.9–86.2%) and 75.7% specificity (95% CI, 71.8%79.4%) at the predetermined cutoff score of 17. The best performance was again in diagnosis of cirrhosis as the algorithm had negative predictive values that ranged from 92.5% to 94.7% in the validation cohorts; it correctly classified 90.0% of F0 samples, 75.0% of F1 samples, 77.4% of F3 samples, and 94.4% of F4 samples [38].

During the last 15 years, a large number of minimal invasive blood testing has been developed. It is important to consider that all serum biomarkers assessing liver fibrosis perform better in late phases than in early stages. The most promising serum biomarkers implemented in clinical trials focusing on emerging treatments are below.

### 4.2. Emerging Minimally Invasive Blood Markers in NASH Diagnosis

The Enhanced Liver Fibrosis (ELF) panel includes serum markers for hepatic metabolism not routinely available (Table 1). ELF was found to be accurate in predicting advanced fibrosis in adult and pediatric patients with NAFLD [39]. The simplified ELF score excluded the age and performed similarly in predicting fibrosis in NAFLD with an AUROC of 0.90. The addition of 5 parameters—BMI, platelets, presence of diabetes mellitus (DM), albumin and AST/ALT ratio—improved the AUROC to 0.98 [40]. ELF can be used as a screening test for advanced fibrosis and is able to reduce the need for liver biopsy of 60% [41].

Pro collagen III (Pro-C3) levels are the expression of extracellular matrix turnover (ECM). A non-epitope-specific antibody—able to bind to protease specific cleavage site of collagen fragments—measures the terminal peptide of procollagen III and, indirectly, active fibrogenesis [42]. It is able to discriminate simple fatty liver from NASH and correlates with severity of steatohepatitis (AUROC 0.85–0.87) and stage of fibrosis with AUROC 0.86 (95% CI 0.78–0.94) [43]. In a large international cohort of patients with biopsy-confirmed diagnosis of NAFLD advanced fibrosis, its performance when included in panels with age, BMI, Type 2 Diabetes and platelets (FIBC3) or when combined with age, T2DM, and BMI (ABC3D) was evaluated. ProC3 showed an AUROC of 0.83 and 0.81 versus 0.76 of FIB-4 in identifying patients with ≥F3 when FIBC3 score was >3 and ABC3D was ≥0.4 [44]. These models, at variance with ELF, include only a single variable that is not routinely evaluated.

Genfit NASH NIS4 is an “in vitro” diagnostic test for NASH. It was developed in the “GOLDEN” Elafibranor Phase II trial and internally validated in a first set of 467 patients screened for “RESOLVE-IT” Phase III trial. NIS4 is used with a single, high-value cutoff correlating to a diagnostic PPV > 80% [45].

An “in vitro” diagnostic algorithm combining 25 different molecular (triglycerides) markers plus BMI, the OWLLiver, has been developed based on two sequential analyses for discrimination between normal liver and NAFLD and between NASH and simple steatosis [46]. Performed on a small amount of plasma by a Liquid Chromatography (LC)-Mass Spectrometry (MS) system, it is a commercial test indicated for adults with BMI > 25 suspected of having NASH. In order to establish the risk of the patients to present NAFLD, NASH, or no NAFLD, this diagnostic method measures two panels of triglycerides in serum and performs a combined analysis with the relative intensities of those triglycerides and the body mass index (BMI) of the patients [47]. This test was initially performed in an original cohort of 467 patients with NAFLD (90 normal liver, 246 NAFL, and 131 NASH). Categorizing patients by their BMI improved the power of the method. Subsequently, the algorithm was validated in a separate cohort of 192 patients (7 NL, 109 NAFL, and 76 NASH). In the latter independent blinded cohort, where the additional risk of high BMI was not predetermined, the AUROC was 0.79.

While the performances of minimally invasive markers of fibrosis is validated using liver histology as a reference standard, noninvasive imaging-based modalities including Transient Elastography (TE), and UltraSound (US)-based elastography have been extensively studied for the assessment of fibrosis or cirrhosis in comparative studies mainly based on patients with viral hepatitis. Among the emerging methods, Magnetic Resonance Elastography is a promising technology.

### 4.3. Emerging Noninvasive Imaging Markers in NASH Diagnosis

TE measures the velocity of an elastic shear wave via propagation of ultrasound waves through the liver (Table 1). The probe utilizes pulse-echo ultrasound to follow the propagation of the shear wave to measure velocity (m/s) and to provide a liver stiffness measurement [48]. Tissue stiffness is related to the degree of fibrosis; the stiffer the tissue, the faster the shear wave propagates. However, TE has some limitations: it cannot be used if there is significant fat or fluid between the chest wall and the liver; it is associated with failures or unreliable results in nearly 20% of patients, particularly in people with obesity. It should be interpreted with caution in case of high transaminases levels, sinusoidal congestion, and extrahepatic cholestasis. Furthermore, elastography measures have been shown to carry considerable variance [49]. The measurement procedure is considered to have failed when no value is obtained after 10 attempts. An examination is considered valid if ≥10 valid measurements are obtained and the interquartile range (which reflects the variability of measurements) is <30% of the median liver stiffness measurement [50]. The results are expressed in kilopascals ranging from 1.5 to 75 kPa, with normal values near 5 kPa. The summary AUROC values using FibroScan M and XL probes for diagnosing advanced fibrosis are 0.88 and 0.85, respectively. The XL probe designed for obese patients produces similar diagnostic accuracy as the M probe.

Point Shear Wave Elastography (pSWE) also known as Acoustic Radiation Force Impulse imaging (ARFI) is an ultrasound-based elastography technique that involves mechanical excitation of tissue using short duration acoustic pulses. Pulses generate shear waves, leading to localized micrometer-scale displacement in liver tissue [51]. Described in many studies as comparable to TE, like TE, it is able to diagnose F4 more accurately than F3 stages. However, pSWE has not been extensively evaluated for NASH diagnosis. Large-scale studies are needed to determine the effects of the narrow measurement range and discrepancies between right and left liver lobe readings sometimes observed.

MRE, like ultrasound-based techniques, is able to determine liver stiffness through the analysis of mechanical waves transmitted through liver tissue. MRE uses a modified phase-contrast method to image the propagation of the shear wave in the liver and is not influenced by the body habitus [52]. It is the most accurate fibrosis test, yet its use is limited by costs, length of the exam, and availability [53]. In a meta-analysis of nine studies involving 232 NAFLD patients, overall MRE was able to detect fibrosis with a high level of accuracy independently of liver inflammation and BMI with AUROC values of 0.86–0.91 for all stages of fibrosis [53]. 

Multiparametric MRI: In the past few years, a multiparametric magnetic resonance technique (LiverMultiScan, Perspectum Diagnostics, Oxford, UK) that includes T1 mapping for fibrosis and inflammation imaging, T2 mapping for liver iron quantification, and magnetic resonance spectroscopy (H-MRS) for liver fat quantification has been implemented [54]. As high iron levels in the presence of fibrosis can lead to “pseudo-normal” T1 values, the development of iron-corrected T1, able to remove iron confounding effect, makes this technique largely applicable [55]. Further validation studies are needed.

## 5. Genetic Biomarkers

NAFLD is considered a complex disease trait with relevant interactions between genetics and environment. The genetic component of NAFLD has emerged in wide genome association studies conducted in recent years. Genetic information acquired by these studies has helped in improving understanding of disease pathogenesis. Two genetic variants (rs738409 and rs58542926), located in the Patatin-like phospholipase domain containing 3 protein (PNPLA3) and Transmembrane 6 superfamily member 2 (TM6SF2) hepatic steallate variants, associated with increased fat content have been identified [56,57]. They are associated with about 3.3% and 2% risk of NAFLD. However, their accuracy in predicting disease is not different from that of other noninvasive biomarkers.

Subsequently, an association of the rs641738 C>T genetic variant in the MBOAT7-TMC4 locus with increased risk of the entire spectrum of NAFLD was also shown [58].

Despite the important impact of these genetic variant discoveries, specific DNA sequence variation, including single nucleotide polymorphisms (SNPs), identify individuals at risk of NAFLD, specificity, sensitivity, and positive and negative predictive values to support their clinical implementation [59]. The incorporation of genetic markers into predictive scores including routine clinical testing has also been explored [60].

Predisposition to progressive NAFLD has also been shown strongly influenced by genetic heritability, but again, the risk effect of the common missense SNP rs738409 in PNPLA3 and SNP rs58542926 in TM6F2 (minor allele frequencies (MAF), the frequency at which the second most common allele occurs in a population, were 0.38 and 0.073, respectively) is not high and even lower when the MAF of TM6SF2 polymorphism (MAF 0.07) is considered.

In summary, inclusion of these genetic variants alongside routine clinical parameters results aiming at predicting the presence of the disease and the probability of progression is limited by an accuracy comparable to that of existing biomarker scores that combine clinical and routine laboratory variables.

## 6. OMICS-Based Markers

The development of modern mass spectroscopy (MS) together with highly efficient technologies has favored the identification of novel biomarkers of NAFLD and NASH through very rapid measurement of thousands of metabolites. In addition, advancements in sequencing and expression profiling technologies and the application to animal models highlighted the role of the genome by showing, for example, that in C57/BL6 mice following high fat diet, 309 genes had altered expression profile, as compared to mice following normal diet [61].

Proteomic technologies allow measurements of large number of proteins in a small serum sample, but a platform for the measurement of those impacting NAFLD is not available. In 98 patients that underwent bariatric surgery, Younussi et al. [62] identified 12 protein peaks with significant differential expression by severity of NAFLD/NASH diagnosis; however, these may not represent the general population of NAFLD patients. In another study in obese patients, Charlton et al. showed that 9 proteins were expressed with differential abundance between study groups [63]. Finally, in a study published in 2010, using a label-free quantitative proteomics approach (LFQP), Bell et al. studied 1738 proteins. Significant changes in 605 proteins were observed when serum samples of control subjects and NAFLD patients—nonobese in the majority of cases—were compared [64]. By contrast, since no significant difference was observed between simple steatosis and NASH subgroups, the identification in the serum of systemic markers of initial mild NASH might be more challenging. All in all, 55 of 605 proteins had different expression between the simple steatosis and NASH F3/F4 group and 15 proteins useful as biomarkers were identified. This serum panel was also shown useful in distinguishing NAFLD patients from patients with drug-induced liver injury [65]. Yu et al. used proteomics to demonstrate that higher baseline hemoglobin values are associated with the development of NAFLD in a prospective group of 6944 subjects [66].

Examining patients with different stages of NAFLD, transcriptomic provided evidence of upregulation of cell survival and liver regeneration genes and of downregulation of defense mechanisms against oxidative stress in early stages of NASH. Upregulation of hepatoc steallate cells, inflammation, fibrogenesis, and detoxification pathways was shown in late stages [62].

Metabolomics have also been used to differentiate metabolic subtypes of human NAFLD. Analyzing the serum metabolic profiles of patients with NASH or with simple steatosis and healthy controls, pyroglutamate showed a promising 82% accuracy rate in distinguishing NASH from simple steatosis groups [67]. Serum metabolomics have identified a panel of biomarkers characterized by marked changes in bile salts and in biochemicals related to glutathione in subjects with nonalcoholic fatty liver disease. Analyzing this pattern, it was possible to distinguish healthy individuals from NASH or NAFLD but not NASH from NAFLD patients [68]. In a small recent study, aiming at identifying the bile acid metabolome in NASH, in the postprandial period, patients with initial NASH showed an increase in taurine and glycine-conjugated primary and secondary bile acids. Progressive decrease towards depletion of secondary bile acids was experienced with the progression to NASH and cirrhosis [69].

Lipidomic appears a promising strategy in NAFLD: beside the studies on bile acid biomarkers, substantial alterations in multiple plasma lipid species across the NAFLD spectrum have been evaluated. Alterations can be related to short chain fatty acid (SCFA) and branched chain amino acid (BCAA). A large study on 679 patients who underwent liver biopsy or MS discovered and validated a signature comprising three lipid molecules. However, results showed modest accuracy in predicting NAFLD (AUROC 0.71–0.74) [70]. In addition to the previously reported study by Iruarrizaga-Lejarreta et al. [46] performed in obese patients, another more recent study of 318 patients who underwent liver biopsy used a model combining genetic, clinical, lipidomic, and metabolomics markers to derive a score (NASH ClinLipMet). Considering this score, it can be possible to identify patients with NASH with an accuracy (AUROC) of 0.86–0.88, and the result is not affected by statin medications or degree of obesity [71]. This score identifies NASH patients with accuracy higher than MS-based profiling alone. Despite these results, complexity and laboratory expertise required for score calculation currently limit its utilization. Notably, the metabolomics alterations in this study include changes in circulating levels of BCAA and essential amino acids as glutamate and serine.

It should be considered that complicated methodologies involved in omics platforms prevent widespread application beyond the research setting.

## 7. Emerging Circulating Biomarkers

Circulating extracellular vesicles (exosomes and ectosomes) contain various cellular molecules such as proteins, mRNA, miRNAs, and DNA and can serve as biomarkers in NAFLD and NASH [72]. Following their release into the intercellular space, ectosomes bind to recipient cells and deliver their informative cargo [72]. The recipient cells may then undergo epigenetic reprogramming and subsequent phenotypic alterations according to the molecular information received [72]. In NAFLD patients, Kornek et al. using a fluoresce-activated cell sorting (FACS) observed an increase in ectosomes in surface markers from monocyte and natural killer and a decrease in neutrophils and leuco-endothelial cells. Production of exosomes and other extracellular vesicles are increased in patients with NASH [73]. Recently, it has been suggested that a specific protein signature in blood extracellular vesicles may be used to diagnose NASH noninvasively [74]. A role for exosomes in the cross talk between hepatocytes and hepatic stellate cells has been hypothesized. Lipotoxic fatty acid-injured hepatocytes produce exosome-like vesicles, which are then taken up by hepatic stellate cells, leading to fibrogenic activation. In transgenic mice, increased levels of CD10 protein in urinary exosomes was associated with steatosis and fibrosis [75].

Besides proteins, other extracellular vesicles-linked biomarkers such as circulating nucleic acids either DNA fragments—usually defined cell-free DNA (cfDNA)—or RNA have been identified. The current focus on cfDNA is on specific characteristics rather than on quantitation. Methylation is one of these characteristics. Hardy et al. showed an increase in cfDNA methylation at the PPARgamma gene promoter in a cohort of patients with NAFLD as compared to healthy controls [76]. This is an important requirement for HSC activation and appears correlated with fibrosis progression in NAFLD. However, the preliminary results indicate substantial lack of specificity as this correlation has been observed also in HBV chronic infection [76].

Cell free noncoding RNA comprises long (lncRNA) and short (miRNA) species of RNA. The lncRNAs are cell-specific molecules with a length of >200 bp and with several of the theorical essential characteristics of good biomarkers, including accessibility, although it is difficult to process them and to obtain accurate measurements [77,78]. Recent in vitro studies reported aberrant expression of lncRNA during HSC activation, and this was confirmed in two in vivo studies examining liver tissue of NAFLD and NASH patients [79,80,81]. In biopsy-proven NAFLD patients with disease of different severity, another small study showed difference in the expression profile of patients when compared to normal liver [82].

The miRNAs, utilized for intercellular signal transduction, are small noncoding microRNAs with epigenetic functions able to transcriptionally regulate gene expression [83]. MiRNAs contribute to the pathogenesis of NAFLD/NASH at various levels of disease development and progression and probably are the most extensively studied epigenetic modifications in NAFLD. In particular, miR-122 has a well-established role in lipid metabolism and was shown upregulated in NASH in comparison to NAFLD [83]. In NASH, the exosome-packaged liver specific miR-122 increases over time and correlates with histological severity. Some studies have demonstrated that, in the liver of patients with NASH, miR-122 is downregulated [83]. Using immunoprecipitation, it was shown that, in NASH, the low intrahepatic levels of miR-122, rather than a real downregulation, may be a consequence of the increased rate of release into the circulation [84]. It can be hypothesized that hepatocyte-derived exosomes mediate miR-122 sensitization of macrophages to inflammatory signals [82]. Other miR such as miR-34a concentrations were significantly higher in NAFLD patients [85]. A specific signature including various miRNAs has been recently identified as able to discriminate between ASH, NAFLD, NASH, and cholestatic disease [83].

These approaches are all promising, although their implementation may currently be problematic since studies related to cfDNA are limited by both the very low concentrations and the high fragmentation and studies related to lncRNA may be limited by small sample size and lack of standardized protocols.

## 8. Microbiota-Related Biomarkers

Profiling of gut microbiota and metabolites associated with NAFLD, NASH, and fibrosis have been explored as attractive alternatives to noninvasively diagnose early stages of disease. Dysbiosis characterized by reduction of phyla Firmicutes, increase of phyla Proteobacteria, and reduction of *Ruminococcus* at both the genus and species levels have been associated with NASH and fibrosis in several studies in humans [86].

Studying the stool microbiome and serum metabolome of a well-characterized prospective cohort of 86 patients with biopsy-proven NAFLD, 37 species associated with advanced fibrosis were identified [87]. This evidence enabled the development of an algorithm that could predict advanced fibrosis with a high degree of accuracy (AUROC 0.936). Subsequent identification of serum metabolites predicted by gut bacteria functional analysis demonstrated differential levels of 11 amino acids and metabolites involved in nucleoside and carbon metabolism, suggesting that a serum test based on gut microbiome profiles could be a useful marker in the future.

## 9. Conclusions

**Figure 1:**
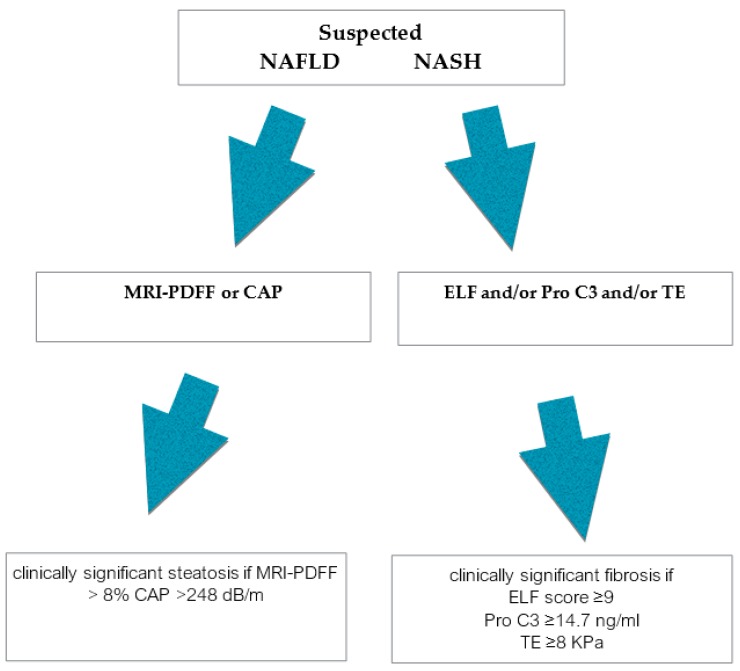
Proposed diagnostic algorithm based on a selection of currently available non- or minimally invasive markers of NAFLD and NASH.

The key to a successful prevention program depends on the early identification of high-risk individuals by measuring a number of specific biomarkers. Presently, as strategies of treatment for NASH patients at risk of progression are implemented, biomarkers are essential for screening and identification of treatment responses. Given the plethora of non- or minimally invasive markers, this review focused only on well-known or recent tools to provide a reference for the use of those biomarkers that perform better in addressing different questions regarding NAFLD or NASH diagnosis or fibrosis progression (Figure 1). Noninvasive imaging techniques such as MRI are evolving at increasing pace, and MRI-PDFF currently provides early diagnosis and prognostic information on NAFLD but it is not largely available. Serum biomarkers for fibrosis diagnosis in NASH perform better in excluding advanced fibrosis and cirrhosis rather than accurately diagnosing fibrosis stages. Procollagen C3 levels permit to discriminate between patients with or without histological diagnosis of NASH and a relatively linear relationship with the grade of NASH. Imaging methodologies and, in particular, MRE are accurate although limited by costs and duration of the exams. Emerging OMICS markers may be promising in the early identification of patients at risk of progressing to advanced fibrosis. However, their accuracy is limited by their challenging methodological implementation. In conclusion, it is not possible to accurately differentiate NAFLD from NASH and NASH of different severity and, consequently, to select the ideal candidate for experimental trials by using one single marker. A combination of the best performing biomarkers needs to be adopted in ongoing clinical trials on novel therapeutic compounds.

## Figures and Tables

**Table 1:** Various emerging modalities for nonalcoholic steatohepatitis (NASH) minimally invasive/noninvasive diagnosis: (A) blood clinical biochemistry liver function tests with liver histology as comparator; (B) physical tests US-based or not.

A. Blood Liver Funtion Tests	Parameter Measured	Pros	Cons	AUROC
ELF panel [39]	Hyaluronic acid (HA), Tissue inhibitor metalloproteinase 1 (TIMP1), and Aminoterminal peptide of procollagen 3 (PIIINP).	Feasible in large number of subjects Good outcome correlation	Commercial test not routinely available	0.93 in adults 0.99 in pediatric patients
Pro-C3 [42]	Pro collagen III	Able to discriminate simple fatty liver from NASH and different stages of fibrosis	Commercial test	0.86
NASH NIS4 [44]	MicroRNA 34a-5p; alpha2 macrogobulin (A2M), Haemooglobin A1c (HbA1c), and Chitinase-3-like protein 1 (CHI3L1 also known as YKL40)	This tool can enrich the selection of patients—candidate to experimental trials—with active NASH and significant fibrosis	Commercial test; performances might vary according to the baseline characteristics of the studied population	0.82
Lipidomic serum test § (OWLiver) [45]	Two subsequent analyses of 11 and 20 triglycerides panel to be used in adults with BMI > 25	Able to discriminate normal liver form NAFLD and NAFLD from NASH	Commercial test performed in a centralized laboratory	0.79 or 0.81 (according to inclusion or exclusion of patients with glucose >136 mg/dl)
**B.US-Based Physical Tests**	**Parameter Measured**	**Pros**	**Cons**	**AUROC**
TE [47,48]	Liver stiffness	Short processing time and outpatient clinic setting	Measurement failures reported in up to 20% and XL probe required in obese patients	0.95 for F4 0.93 for F3 0.84 for F2 fibrosis
Point shear wave elastgraphy (ARFI) [49]	Liver stiffness	Short processing and outclinic setting	Quality criteria not well defined, lack of large-scale studies	0.78–0.89 for F4 0.74–0.97 for F3 0.70–0.83 for F2 fibrosis
**B. Not US-Based physical tests**	**Parameter Measured**	**Pros**	**Cons**	**AUROC**
MRE [50,51]	Liver stiffness	Not influenced by BMI and inflammation	Long processing, expensive, and not largely available	0.88–0.97 for F4 0.89–0.96 for F3 0.86–0.89 for F2
LiverMultiScan (multiparametric resonance) [52]	Fibrosis and inflammation mapping	Quick and no contrast agent required	Further validation studies required	0.85 for F4

§ compared to histology.

## Abbreviations

A2M	alpha2macroglobulin
AMEe	S-Adenosylmethionine
APO-F	Plasmatic apolipoprotein
AUROC	Area Under the Receiver Operating Characteristics
ALT	alanine aminotransferase
AST	aspartate aminotransferase
BCAA	Brached chain aminoacid
BMI	Body mass index
CAP	Controlled attenuated parameter
CHI3L	chitinase 3-like protein 1
CK18	Cytokeratin 18
ELF	Enhanced Liver Fibrosis panel
ECM	Extracellular matrix turnover
FIB-4	Fibrosis Index-4
F3/F4	Fibrosis stage 3 /4
FGF21 gene	Fibroblast growth factor gene
FLI	Fatty liver Index
FXR	Farnesoid X receptor
GGT	gammaglutamil transferase
HA	Hyaluronic acid
HIS	Hepatic Steatosis Index
H-MRS	Magnetic resonance spectroscopy
KPa	KiloPascal
IL-8	interleukin 8
LC-MS	Liquid chromatography-Mass spectrometry
LFQP	label free quantitative proteomics approach
LncRNA	long non codingRNA
MAF	Minor allele frequency
MBOAT7	Membrane-bound O-acyltransferase domain-containing protein 7
MRI-PDFF	Magnetic resonance imaging-proton density fat fraction
MRE	Magnetic Resonance elastography
MRI	Magnetic Resonance Imaging
miRNA	microRNA
NAFLD	Non alcoholic fatty liver disease
NLFS	NAFLD Liver Fat score
NASH	Non alcoholic steatohepatitis
NASHTest	Non alcoholic steatohepatitis test
NPV	negative predictive value
PLT	platelets
PNPLA3	Patatin-like phospholipase domain-containing protein 3
PPV	Positive predictive value
pSWE	Point shear wave elastography
Pro-C3	Pro-ollagen III
sCD	soluble macrophage activation marker
T2DM	type 2 diabetes mellitus
TE	Transient Elastography
TIMP1	Metallopeptidase inhibitor 1
TGs	tryglicerides
TM6F2	Transmembrane 6 superfamily mamber 2
TNF	tumor necrosis factor
TyG	Triglyceride and Glucose Index
US	ultrasound
VAI	Visceral Adiposity Index
